# Cattle In Vitro Induced Pluripotent Stem Cells Generated and Maintained in 5 or 20% Oxygen and Different Supplementation

**DOI:** 10.3390/cells10061531

**Published:** 2021-06-17

**Authors:** Brendon Willian Bessi, Ramon Cesar Botigelli, Naira Caroline Godoy Pieri, Lucas Simões Machado, Jessica Brunhara Cruz, Pamela de Moraes, Aline Fernanda de Souza, Kaiana Recchia, Gabriela Barbosa, Raquel Vasconcelos Guimarães de Castro, Marcelo Fábio Gouveia Nogueira, Fabiana Fernandes Bressan

**Affiliations:** 1 Department of Veterinary Medicine, Faculty of Animal Science and Food Engineering, University of São Paulo (USP), Pirassununga 13635-000, Brazil; brendon.bessi@usp.br (B.W.B.); nairagodoy@usp.br (N.C.G.P.); lucas.machado@unifesp.br (L.S.M.); jessica.brunhara.cruz@usp.br (J.B.C.); pamela.moraes@usp.br (P.d.M.); alinsouza@usp.br (A.F.d.S.); kaiana.recchia@usp.br (K.R.); gabriela2.barbosa@usp.br (G.B.); raquel.castro@unesp.br (R.V.G.d.C.); 2 Department of Pharmacology, Institute of Biosciences (IBB), São Paulo State University (UNESP), Botucatu 18618-689, Brazil; 3 Department of Animal Reproduction, Faculty of Veterinary Medicine and Animal Sciences, University of São Paulo (USP), São Paulo 05508-270, Brazil; 4 Department of Pathology, Reproduction and One Health, Faculty of Agricultural and Veterinary Sciences, São Paulo State University (UNESP), Botucatu 14884-900, Brazil; 5 Department of Biological Science, School of Sciences, Humanities and Languages, São Paulo State University (UNESP), Assis 19806-900, Brazil; marcelo.fabio@unesp.br

**Keywords:** oxygen, acquisition of pluripotency, bovine iPSCs, pluripotent state

## Abstract

The event of cellular reprogramming into pluripotency is influenced by several factors, such as in vitro culture conditions (e.g., culture medium and oxygen concentration). Herein, bovine iPSCs (biPSCs) were generated in different levels of oxygen tension (5% or 20% of oxygen) and supplementation (bFGF or bFGF + LIF + 2i—bFL2i) to evaluate the efficiency of pluripotency induction and maintenance in vitro. Initial reprogramming was observed in all groups and bFL2i supplementation initially resulted in a superior number of colonies. However, bFL2i supplementation in low oxygen led to a loss of self-renewal and pluripotency maintenance. All clonal lines were positive for alkaline phosphatase; they expressed endogenous pluripotency-related genes *SOX2*, *OCT4* and *STELLA*. However, expression was decreased throughout the passages without the influence of oxygen tension. *GLUT1* and *GLUT3* were upregulated by low oxygen. The biPSCs were immunofluorescence-positive stained for OCT4 and SOX2 and they formed embryoid bodies which differentiated in ectoderm and mesoderm (all groups), as well as endoderm (one line from bFL2i in high oxygen). Our study is the first to compare high and low oxygen environments during and after induced reprogramming in cattle. In our conditions, a low oxygen environment did not favor the pluripotency maintenance of biPSCs.

## 1. Introduction

The success of reprogramming somatic cells into pluripotent cells, or iPSCs, has long been reported and studied, particularly in humans and mice. This technology has provided researchers with a valuable source of different cell types [1,2,3] to use in regenerative medicine, as well as for biotechnology and reproductive purposes in domestic and wild animals [4].

Pluripotent stem cells are mainly classified into two different states, naïve and primed. Naïve ESCs and iPSCs, mostly observed in murine models, rely on leukemia inhibitory factor (LIF) supplementation to retain pluripotency, whereas primed mouse EpiSC and human ESCs and iPSCs rely on fibroblast growth factor 2 (FGF2) stimulation [5]. Additionally, two small molecules are widely used to improve the pluripotent state by blocking the mitogen-activated protein kinase (MEK) and Glycogen synthase kinase 3-beta (GSKB3) activity pathways; these molecules are known as 2i (2 inhibitors) [6].

iPSCs from large animals are important models for both biomedical and agricultural applications due to their physiological and morphological similarity to humans and, in particular, cattle genetically manipulated by different genome editing tools would provide a powerful platform for the production of modified herds with superior genetic traits for production of reproduction. Indeed, multiple genetic alterations in livestock could be used to improve traits connected with animal production and create large animal models for in vitro and in vivo disease modeling [4,7,8,9].

The generation of livestock iPSCs has been reported, including in sheep [10,11], horses [12,13], pigs [14,15,16] and cattle [17,18,19]. These livestock iPSCs revealed pluripotent characteristics, such as a high proliferation capacity, the expression of pluripotency marks and the ability to generate three germ layers tissues on in vitro and in vivo tests; however, most of the characteristics and protocols are still divergent and often describe either an intermediate or “pluripotency-like” state [20].

Different methodologies have been explored to improve efficiency during the reprogramming process. The in vitro culture environment is one promising field that enables the use of different molecules as inhibitors and supplements and allows the control of oxygen tension. In mouse ESCs, hypoxia conditions (5% oxygen) increased the number of colonies with alkaline phosphatase activity, enhanced cell proliferation and prevented differentiation [21]. Additionally, for mouse and human iPSCs, hypoxia increased reprogramming efficiency by promoting an increased number of colonies [22]. However, livestock iPSCs have not yet been described on low oxygen.

This study aimed to reprogram bovine fibroblasts under different culture conditions and oxygen tensions to generate biPSCs. These were then evaluated through their pluripotency-related transcripts at different passages, immunostaining and in vitro differentiation capacity. As such, we aimed to obtain a better understanding of the failures in the establishment of the pluripotency state in biPSCs that are mainly associated with mechanisms of self-renewal and related to in vitro culture conditions.

## 2. Materials and Methods

All experiments were approved by the Ethics Committee on Animal Experimentation at the Faculty of Animal Science and Food Engineering of the University of São Paulo (CEUA 2192250918 and 3526250717).

### 2.1. Cells and Media

Mouse embryonic fibroblasts (MEFs), bovine fetal fibroblasts (bFFs) and 293FT cells were cultured in complete Iscove′s Modified Dulbecco′s Medium (IMDM; 12200-036, Thermo Fisher Scientific, Waltham, MA, USA) supplemented with 10% fetal bovine serum (FBS; SH30071.03, Global Life Sciences Solutions USA LLC, Marlborough, MA, USA), 1% GlutaMAX (3505006, Thermo Fisher Scientific, Waltham, MA, USA), 1% MEM Non-Essential Amino Acids Solution (11140050, Thermo Fisher Scientific, Waltham, MA, USA) and 1% penicillin/streptomycin (15140122, Thermo Fisher Scientific, Waltham, MA, USA). The biPSCs were cultured in KnockOut DMEM-F12 (12660-012, Thermo Fisher Scientific, Waltham, MA, USA) supplemented with 20% KnockOut Replacement Serum (10828028, Thermo Fisher Scientific, Waltham, MA, USA), 1% GlutaMAX, 1% MEM Non-Essential Amino Acids Solution, 1% penicillin/streptomycin and 3.85 μM β-mercaptoethanol (21985-023,Thermo Fisher Scientific, Waltham, MA, USA) and were either supplemented or not with 10 ng/mL recombinant basic FGF (bFGF, 100-18B, Peprotech, Rocky Hill, NJ, USA), 1000 IU/mL recombinant mouse LIF protein (ESGRO, ESG110, EMD Millipore Corporation, Billerica, MA, USA) and 2i (1 mM PD0325901 and 3 mM CHIR99021, Sigma-Aldrich, St. Louis, MO, USA).

### 2.2. Feeder Cells

MEFs were isolated from 13.5d mouse fetuses and cultured on complete IMDM. Cells were treated with mitomycin (M4287, Sigma-Aldrich, St. Louis, MO, USA) and used as a feeder monolayer at 1 × 10^5^ cells in 6-well dishes. The dishes were treated with 0.1% gelatin (G1393, Sigma-Aldrich, St. Louis, MO, USA) prior to seeding.

### 2.3. Lentivirus Production

Lentiviral production was performed as described before [18]. Briefly, 5 × 10^6^ 293FT cells were seeded in t75 cm^2^ flasks and maintained in IMDM with 10% FBS overnight. The STEMCCA vector (12 μg) containing the murine transcription factors OCT4, KLF4, SOX2 and c-MYC [23] and the auxiliary plasmids (1.2 μg TAT, 1.2 μg REV, 1.2 μg Hgpm2 and 2.4 μg VSVG) were transfected using the Lipofectamine 3000 reagent (L3000-015, Thermo Fisher Scientific, Waltham, MA, USA) following the manufacturer’s suggestions. The media containing the lentivirus particles were recovered at 24–72 h after transfection. The supernatant was filtered (0.45 μm membrane, SCHVU01RE, EMD Millipore Corporation, Billerica, MA, USA) and distributed evenly into ultracentrifuge tubes. The ultracentrifugation was performed at 48,960 G over 90 min and at 4 °C. The supernatant was removed and approximately 200 μL per tube was used to resuspend the lentivirus stock. The lentivirus stock was aliquoted and stored at −150 °C.

### 2.4. Bovine Induced Pluripotent Stem Cells Generation

To reprogram fetal fibroblasts, the cells were seeded 24 h before lentivirus transduction at 2 × 10^4^ per well on a 6-well plate. A fresh medium with lentivirus stock was added to the culture medium supplemented with 8 μg/mL hexadimethrine bromide (polybrene, Sigma-Aldrich, St. Louis, MO, USA. After overnight transduction, the culture medium was replaced by fresh medium. On day 5 after transduction, the cells were harvested with TrypLE (12604-021, Thermo Fisher Scientific, Waltham, MA, USA) and split into plates with feeder layers and iPSC medium supplemented with bFGF or bFGF + LIF + 2i (bFL2i). The culture was performed at two different oxygen levels (5%—low O_2_ tension—and 20%—high O_2_) at 38.5 °C and 5% CO_2_ in a humidified atmosphere. Three clonal lineages were analyzed for each experimental group.

### 2.5. In Vitro Embryo Production

One pool of in vitro produced blastocysts was used as positive control for pluripotency analyses. For that, bovine ovaries were collected from a local abattoir and cumulus–oocyte complexes (COCs) were aspirated from selected follicles (2–6 mm in diameter) using a 21-gauge needle. COCs were incubated in tissue culture medium 199 (TCM-199) supplemented with 10% FBS, 0.2 mM pyruvate, 50 µg/mL gentamicin, 0.5 mg/mL FSH and hCG, 5 IU/mL (Vetecor, Ceva Hertape, Brazil) for 22 h. The in vitro fertilization was performed with frozen–thawed semen from a single Holstein bull. Capacitated sperm were obtained after Percoll gradient (45% and 90%) separation. The fertilization medium was composed of Tyrode’s lactate stock, 50 μg/mL gentamicin, 22 μg/mL sodium pyruvate, 40 μL/mL PHE (2 mM D-penicillamine, 1 mM hypotaurine and 245 μM epinephrine), 5.5 IU/mL heparin and 6 mg/mL bovine serum albumin (BSA). Presumptive zygotes were partially denuded after 18 h and cultured in SOF medium in an incubator with a humidified atmosphere of 5% CO_2_ and 5% O_2_ in air at 38.5 °C for 7 days (insemination is considered as day 0).

### 2.6. Alkaline Phosphatase Activity and Immunofluorescence

Alkaline phosphatase activity was detected with an alkaline phosphatase kit (Leukocyte Alkaline Phosphatase kit, cat. # 86R, Sigma-Aldrich, St. Louis, MO, USA), according to the manufacturer′s instructions. For immunofluorescence, the cells at approximately p20 were fixed with 4% paraformaldehyde (PFA) in Dulbecco’s phosphate-buffered saline (DPBS) for 10 min, permeabilized with 1% Triton X-100 in DPBS at 20 min, blocked for 1 h with 1% bovine serum albumin (BSA, A2153, Sigma-Aldrich, St. Louis, MO, USA) and 0.3% Triton X-100 in DPBS and incubated with primary antibody overnight at 4 °C in blocking solution, anti-SOX2 (S9072-100UG, 1:500; Sigma-Aldrich, St. Louis, MO, USA) and anti-OCT4 (sc-8628, 1:100; Santa Cruz Biotechnology, Dallas, TX, USA). The next day, cells were washed with DPBS and 0.05% tween 20 and incubated with secondary antibodies (Alexa Fluor 568-conjugated goat anti-rabbit IgG (A-11036, Thermo Fisher Scientific, Waltham, MA, USA), and Alexa Fluor 488-conjugate goat anti-mouse IgG (A-11029, Thermo Fisher Scientific, Waltham, MA, USA), for 1 h at room temperature. Then, cells were washed 3× for 10 min each; on the second wash, 10 μg/mL of Hoechst 33,342 was included for 10 min. Samples were mounted on microscope slides with ProLong Gold Antifade Mountant (P36935, Thermo Fisher Scientific, Waltham, MA, USA). Images were captured using an EVOS™ digital inverted microscope (Thermo Fisher Scientific, Waltham, MA, USA).

### 2.7. RNA Extraction and Reverse Transcription

All samples were submitted to total RNA extraction using the Trizol^®^ protocol (Thermo Fisher Scientific, Waltham, MA, USA). After extraction, total RNA samples were quantified with a NanoDrop^®^ (Thermo Fisher Scientific, Waltham, MA, USA). For DNA digestion and reverse transcription, we adjusted the concentration to 1.000 ng RNA per sample. All the samples were subjected to DNA digestion using a DNAse I—Amplification Grade^®^ (18068015, Thermo Fisher Scientific, Waltham, MA, USA). For reverse transcription (RT) of the samples, a high-capacity cDNA reverse transcription kit was utilized (4368814, Thermo Fisher Scientific, Waltham, MA, USA).

### 2.8. Quantitative PCR

For gene expression analysis on biPSCs (generated in 5% and 20% O_2_ conditions and different supplements), samples were collected at five different moments during the reprogramming process (passages 5, 10, 15, 20 and 25). The targets were pluripotent and metabolism-related genes and the exogenous OSKM expression. The reference genes were *ACTB* and *PPIA* [24] and normalization was performed using the geometric means from both. The primers were designed using the Primer-BLAST (NCBI) software based upon sequences available in GenBank (Table 1).

Relative quantification of transcribed genes was analyzed by qPCR using the PowerUP SYBR Green^®^ PCR Master Mix reagent (A25742, Thermo Fisher Scientific, Waltham, MA, USA) in Applied Biosystems QuantStudio 6 equipment. Then, qPCR reactions were run in a volume of 10 μL containing 100 nM of each primer, 1X Power UP SYBR Green, 2.5 μL H_2_O and 1 μL template (four-fold diluted cDNA; 25 ng). Cycling conditions for amplification were as follows: 95 °C for 10 min, followed by 45 cycles at 95 °C for 15 s, 57 °C for 20 s and 60 °C for 40 s. Each sample was analyzed in duplicate for each of the genes. Additionally, ultrapure DNAse and RNAse free water was used as a negative control of the reaction. The relative gene expression was determined by 2^−ΔCq^.

### 2.9. Undirected Differentiation in Embryoid Bodies

Undirected differentiation of bovine iPSCs at p25 through embryoid bodies (EBs) formation was performed in plates treated with agarose 0.1% and iPSCs media without bFGF, 2i, or LIF. After 5 days in suspension culture, EBs were collected for qPCR analysis. EBs were placed on gelatin-coated culture plates and cultured in IMDM medium; EBs were documented 7 days after plating.

### 2.10. Statistical Analysis

Statistical analysis was performed using GraphPad Prism 8 (GraphPad Software, San Diego, CA, USA). Data were tested for normality of residuals and homogeneity of variances using the Shapiro–Wilk test and analyzed. Gene expression on biPSCs lines data were analyzed by two-way ANOVA followed by Tukey’s post hoc test. Gene expression on EBs data were analyzed by one-way ANOVA followed by Tukey’s post hoc test. Differences with probabilities of *p* < 0.05 were considered significant. In the text, values are presented as the mean ± the mean standard error (SEM). All experiments were repeated at least three times unless otherwise stated.

## 3. Results

### 3.1. Influence of Oxygen Tension and Different Pluripotency Inducers during the Reprogramming Process of Bovine Fibroblasts

Colonies were observed on day 14 after transduction and the number of colonies that appeared in one well of each treatment was counted for each experimental group. A higher number of colonies was observed with bFL2i supplementation in both oxygen tensions (0.075 and 0.007%, respectively, for hypoxia and normoxia, meaning that 15 and 14 colonies were observed at D14 after plating 2 × 10^4^ transduced cells per well; Table 2), whereas 0.02% and 0.005% were observed for the bFGF group (4 and 1 colonies observed at D14, respectively, for hypoxia and normoxia).

Three colonies from each experimental group were selected at day 21 post-transduction for expansion and clonal culture. All lines were cultured for 25 passages (±200 days), with the exception of the clonal lines derived from the bFL2i group in hypoxia conditions, which lost its iPSCs characteristics after 8 passages, suggesting a loss of its self-renewal ability and the maintenance of pluripotency.

**Figure 1:**
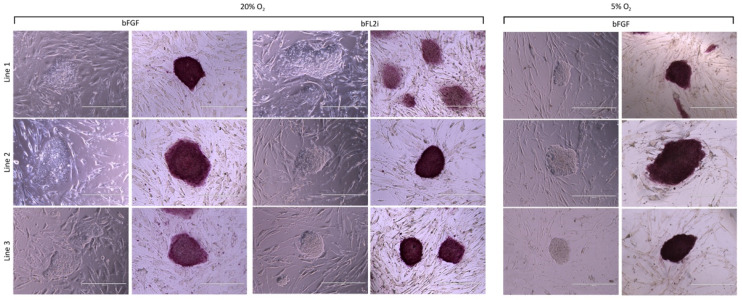
Bovine induced pluripotent stem cells derived in different oxygen tensions (low × high O_2_) and pluripotent supplements (bFGF × bFL2i). All biPSCs colonies generated were small and compact colonies, with clearly defined borders, and were positive for alkaline phosphatase. Magnification 10×, Scale bars: 400 μm.

All biPSCs lines generated in low oxygen or high oxygen tensions were small and compact colonies, with clearly defined borders (Figure 1) and were positive for alkaline phosphatase (Figure 1). Additionally, we did not observe morphological differences between the biPSCs groups.

Immunofluorescence revealed that all lineages were positive for SOX2 and OCT4 (Figure S1); however, both genes are also known to be expressed from the exogenous cassette.

### 3.2. Molecular Profile of biPSCs Generated in Different Oxygen Tensions and Different Supplementations

**Figure 2:**
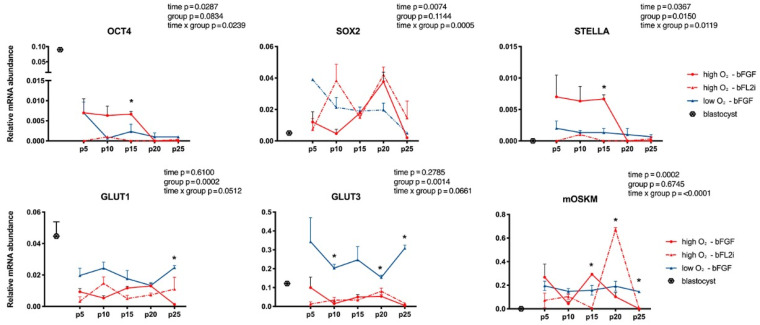
Gene expressions of SOX2, OCT4, STELLA (pluripotent markers), GLUT1 and GLUT3 (metabolism pathways) and mOSKM (exogenous vector) in biPSCs derived at different oxygen tensions (low × high O_2_) and pluripotent supplements (bFGF × bFL2i). Statistical differences in time, group effect and the interaction between time and treatment were observed in each gene. Statistical differences between groups in the same passage are presented with *. The Y axis represents arbitrary units.

The generated biPSCs were analyzed at five different time points during culture: p5, p10, p15, p20 and p25. At initial passages, (p5) no differences were observed. The SOX2 relative gene expression varied throughout all passages (*p* = 0.0074); however, experimental groups did not differ. Relative expression of OCT4 and STELLA increased in the bFGF group that was cultured in high O_2_ compared to low O_2_; in normoxia, bFGF supplementation showed higher expression than bFL2i for these genes. However, both genes decreased after passaging (*p* = 0.0287 and *p* = 0.0367 for OCT4 and STELLA, respectively) (Figure 2).

SLC2A1 and SLC2A3 (also called GLUT1 and GLUT3) presented higher relative expression in hypoxic conditions (*p* = 0.0002 and *p* = 0.0014, respectively). In normoxia, no differences in bFGF x bFL2i supplementation were observed (Figure 2). The exogenous expression of mOKSM was reduced throughout passaging (*p* = 0.0002); however, groups were not different (*p* = 0.6745) (Figure 2).

### 3.3. Undirected Differentiation of biPSCs Generated in Hypoxia and Normoxia Conditions and Different Pluripotent Supplements

**Figure 3:**
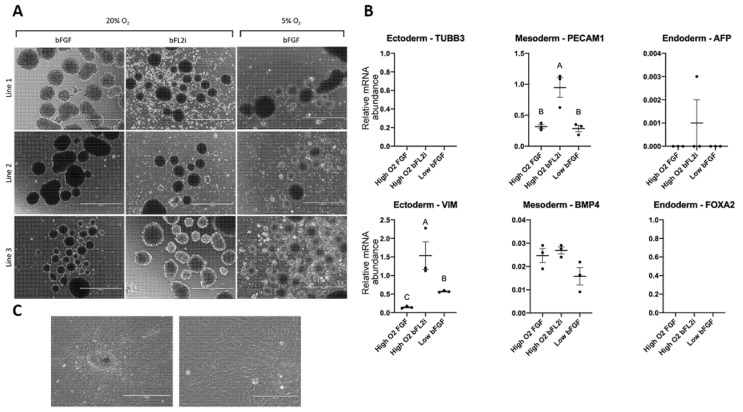
Morphological and gene expression analyses of the embryoid bodies (**A**) and derived culture after EBs dissotiation and undirected differentiation (**B**,**C**). (**A**). All biPSC lines were able to be differenciated in EBs after 5 days in supension culture. (**B**) An analysis of the gene expression showed the genes VIM, PECAM1 BMP4 and AFP; however, TUBB3 and FOXA were observed. Statistical differences are presented by different letters (*p* < 0.05). (**C**) The EBs were able to adhere to the plate and were differentiated into morphologically different cellular types. Scale bars: 400 and 200 μm.

In vitro differentiation of cells was performed via the undirected differentiation of embryoid body (EB) formation. All biPSCs lines generated in hypoxia and normoxia conditions and different pluripotent supplements were cultured in suspension for 5 days without supplementation. All lineages were able to produce EBs (Figure 3A). Additionally, these EBs were evaluated by qPCR regarding ectoderm, mesoderm and endoderm markers.

The relative expression of the ectoderm marker (Vimentin—VIM) and mesoderm markers (BMP4 and PECAM1) was observed for all lines. However, we did not observe the relative expression of TUBB3 and FOXA2, both ectoderm and endoderm markers, respectively (Figure 3B).

Interestingly, we observed the relative gene expression of AFP, an endoderm marker, in EBs derived from biPSCs culture in bFL2i in 20% O_2_ conditions only. Oxygen tension did not seem to affect cell differentiation in our experimental conditions; nevertheless, we observed a higher expression of PECAM1 and VIM on EBs produced from the bFL2i high oxygen tension group (Figure 3B).

Additionally, EBs were transferred to gelatin-coated dishes and cultured for another 7 days with IMDM supplemented with FBS. During the culture, it was possible to identify several different types of cells presenting varying morphologies (Figure 3C).

## 4. Discussion

The generation of bovine in vitro reprogrammed cells has been already reported [17,18,25]. Indeed, it may contribute to the generation of genetically modified herds for optimized milk and meat production, as well as to the production of therapeutic proteins, the generation of animals resistant to several syndromes or diseases and even to biomedical models for basic and applied medicine due to cattle’s more similar characteristics to humans in embryology, size and longevity when compared to rodents [26,27].

Herein, we describe the generation of bovine iPSCs generated in 20% and 5% O_2_. We performed a factorial experimental design of different oxygen tensions (5 and 20%) with different supplements (bFGF and bFL2i) to maintain in vitro bovine reprogrammed cells. In our conditions, we observed the formation of biPSCs colonies in both hypoxia and normoxia conditions after 14 days of transduction, with a higher percentage of colonies formed per transduced cell when using bFL2i supplementation independently of oxygen tension; however, further investigation and replicates must be carried out to confirm such initial observations.

In an elegant review, Nichols and Smith described two different states of pluripotency, naïve and primed; each state was described as having typical colony morphology. Pluripotent stem cells presenting the naïve profile grow as dome-shaped and compact colonies with well-defined borders, whereas primed profiles grow as flat colonies [5]. Indeed, pluripotency maintenance using different supplementation relies on several pluripotency pathways, leading to transcriptional and morphological changes during the reprogramming process. The fibroblast growth factors (FGFs) phosphorylate and activate FGF receptors (FGFRs), which leads to protein kinase C (PKC) signaling, regulating cell self-renewal, metabolism, survival, proliferation and differentiation [27]. LIF inhibits differentiation activity in iPSCs and stimulates self-renewal, directing them to pluripotency by acting in three ways: STAT3, PI (3)-kinase and MAPK [28]. Moreover, 2i differentiation inhibitors (MEKi: PD0325901 and GSK3i: CHIR99021) inhibit both MAPK and GSK-3 signaling, regulating the maintenance of naïve pluripotency by inhibiting the fibroblast growth factor receptor (FGFR) and repressing DNA methyltransferase (Dnmt3) expression [29].

The first reports of bovine iPSCs (in 2011–2012) differed regarding colony morphology; when biPSCs showed dome-shaped [17,30] or flat colonies [25,31], authors did not classify those lines as belonging to naïve or primed profiles. More recently, Kawaguchi et al. [32] demonstrated the differences in the morphology of colonies with different supplementations; bFGF + LIF lineages presented a flattened shape, whereas lineages cultured with PD0325901 (MEK pathway inhibitor) changed to a dome-shaped morphology. Additionally, a higher efficiency in colony generation was reported by using a combination of bFGF + LIF + 2i + forskolin.

Furthermore, oxygen tension can modulate different pathways, including those related to the maintenance of pluripotency. During the maintenance of human embryonic stem cells in culture, Ezashi et al. [33] suggested that the hypoxia environment was able to preserve the pluripotency of these cells and diminish the differentiated cells in culture; initial studies in iPSCs described the improvement of reprogramming efficiency in hypoxia [22,34]. Conversely, in this study, hypoxia tension could not maintain the pluripotent state of biPSCs when they were supplemented with bFL2i and these cells differentiated before 10 passages.

De Castro et al. [35] demonstrated that hypoxic conditions did not improve the reprogramming efficiency in equine iPSCs, as has previously been described in humans and mice; however, they also described different profiles of eiPSCs generated in hypoxic compared to normoxia. Indeed, the reprogramming of iPSC cells in a low oxygen atmosphere has not yet been studied in many species, including cattle. Under our experimental conditions, significant differences in the biPSCs colonies morphology were not observed, including when they were derived in either low or high O_2_ or when they were derived in different supplementations.

The relative gene expression of pluripotency targets was evaluated and, in our conditions, no differences were observed in initial passages (p5). We observed the endogenous expression of *SOX2*, *OCT4* and *STELLA* (also known as Dppa3); for these genes, we observed relative expression decreasing in early to late passages, which could be influenced by a freeze–thaw cycle (not shown) or differentiation due to exogenous vector silencing at 20% O_2_.

Lengner et al. [36] described the inefficiency of different oxygen tensions for promoting significant changes in the expression of core pluripotency genes *OCT4*, *SOX2* and *NANOG* in human ESCs. In this work, we did not observe *SOX2* and *OCT4* expression changes when cultured in hypoxia or normoxia conditions. Another possible explanation for the pluripotency core transcription inefficiency is related to oxygen levels—lower oxygen levels (2% or less of oxygen) could potentially promote pluripotency core transcription regulation.

Great advances have been made in understanding pluripotency acquisition during the reprogramming process, which is itself considered to be a highly heterogenous process. For example, the mesenchymal-to-epithelial transition has long been reported as an important step for pluripotency acquisition [37]. More recently, Guo et al. 2019 demonstrated two other important pathways important to determine the reprogrammed fate through mouse reprogramming. Using the single-cell resolution analysis, they showed that Klf4 transcription factor contributes to the non-reprogrammed *Cd34*+/*Fxyd5*+/*Psca*+ keratinocyte-like fate and that IFN-γ impedes the final transition to chimera-competent pluripotency along the reprogrammed cells [38]. Nonetheless, a detailed description of a time-lapse gene expression analysis during reprogramming and pluripotency maintenance (comparing passages) in other species is still lacking in the literature. Several species-specific differences could influence the response of oxygen tension and the reprogramming process. Under our conditions, we maintained pluripotent-like cells for at least 25 passages in both oxygen conditions.

In low O_2_ conditions, *GLUT1* and *GLUT3* expression was upregulated in our biPSCs. *GLUT3* demonstrated a higher affinity for glucose than *GLUT1* and its high turnover makes it an efficient transporter [39]. Additionally, *GLUT3* is the functional transporter for maternal glucose in mouse embryos and *GLUT3* expression is required for blastocyst formation [40]. Somewhat similar to our result, Harvey et al. [41] described the same upregulation in *GLUT1* expression in bovine blastocysts cultured under 2% oxygen and blastocysts cultured under 7% and 20% oxygen. Additionally, in in vitro hypoxia conditions (2% oxygen), *GLUT1* expression was similar to that of in vivo-derived embryos. Controversially, hypoxia conditions (5% oxygen) decreased *GLUT3* expression in equine iPSCs and human embryonic stem cells [35,42]. These controversial results demonstrate the different effects of hypoxia on metabolism pathways between species.

**Figure 4:**
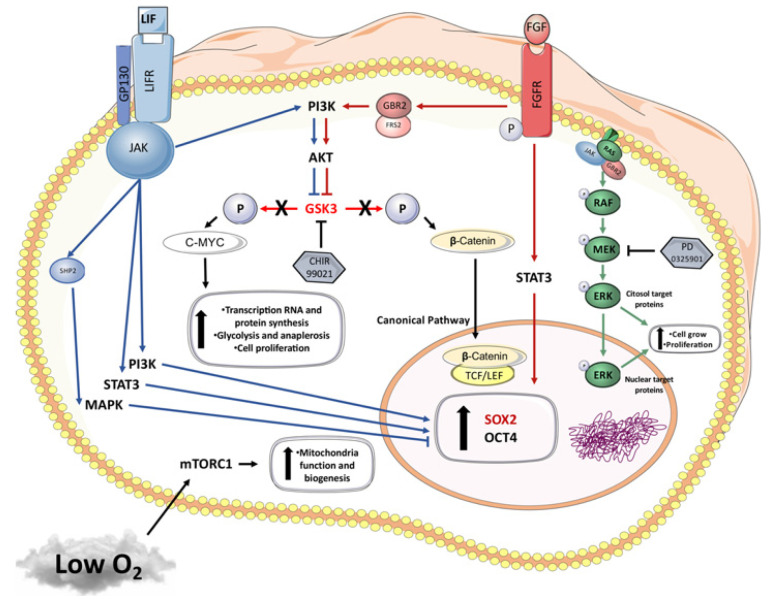
Graphical hypothesis of molecular pathways involved on the cell signaling process during the reprogramming process using different inhibitors of differentiation and low oxygen tension, based on our results, as well as literature data [28,43,44,45,46,47].

Under our conditions, we hypothesize, based on literature data, the molecular pathways (Figure 4) being modulated and discuss the possible action of CHIR99021 in its inhibition of the GSK3 pathway and rising beta-catenin nuclear concentration, therefore leading to *OCT4*, *SOX2* and *NANOG* expression. We suggest that the resultant *C-MYC* expression increase, an essential gene for increasing proliferation and work in the reprogramming process, together with low oxygen tension, may inhibit the mTORC1 pathway, thus decreasing biogenesis and mitochondrial function and potentially explaining the increase in *GLUT3* once glycolysis becomes the only option for cells with low oxygen tension.

It is important to stress that most of the iPSC lines established in non-rodent species, especially livestock species, are dependent on continuous transgene expression to maintain their pluripotency [20]. In bovine iPSCs, Kawaguchi et al. [32] described the same characteristics of naïve-type iPSCs under the control of transgenes; after Dox removal, biPSCs readily changed their morphology and no longer expressed both exogenous and endogenous pluripotent genes. More studies are necessary to optimize culture conditions for the establishment and maintenance of ESCs and iPSCs, while naïve, intermediate and primed states seem to vary among species, which may prove a challenge for solving these issues in livestock species.

In conclusion, one biPSCs line was positive for differentiation in all three embryonic tissues. This lineage was cultured at a high O_2_ tension and with bFL2i. These undirected differentiation results may present an incomplete ability of our EBs to express ectoderm, mesoderm and endoderm markers after the reprogramming process in our conditions, which is probably due to an insufficient period of in vitro differentiation or transgene expression. Indeed, we underline that, however the hypoxia condition was described in mice cells as an enhancer to promote differentiation in the endoderm lineage [45], the same effects were not found in bovine EBs derived from biPSCs that were produced and cultured in low O_2_ conditions.

## 5. Conclusions

The acquisition and maintenance of pluripotency in vitro offers an important tool for improving knowledge on early development. For the first time, our findings show biPSCs characteristics when they are generated under low oxygen tension. In our conditions, oxygen tension may lead to less pronounced differences in in vitro pluripotency maintenance in biPSCs when compared to previous reports on humans and mice. Herein it was evident that bFGF combined with LIF and 2i did not favor pluripotency maintenance at 5% O_2_. In addition, our findings may contribute for demonstrating the ways in which a combination of oxygen and different supplementation sets impact bovine iPSCs.

## Figures and Tables

**Table 1:** List of primer sequences, transcript IDs and product sizes used to characterize pluripotency, metabolism and differentiation.

Primer	Transcript ID	Foward (5′ → 3′)	Reverse (5′ → 3′)	Product Size (bp)
ACTB	NM_173979.3	CAGCAGATGTGGATCAGCAAGC	AACGCAGCTAACAGTCCGCC	89
PPIA	NM_178320.2	CATACAGGTCCTGGCATC	CACGTGCTTGCCATCCAA	107
OCT4	NM_174580.2	GCAAACGATCAAGCAGTGACTAC	GGCGCCAGAGGAGAGGATACG	93
SOX2	NM_001105463.2	ATGGGCTCGGTGGTGAAGT	TGGTAGTGCTGGGACATGTGA	178
STELLA	NM_001111109.2	AGTGAGCGGAGGTACAGGAT	TCGCACTCTTGATCGAATCTCA	132
GLUT1	NM_174602.2	ATCCTCATTGCCGTGGTGCT	ACGATGCCAGAGCCGATGGT	133
GLUT3	NM_174603.3	CGCCTTTGGCACTCTCAAC	GCACTGGATGATGGCTGGTAA	88
TUBB3	NM_001077127.1	GGATAGACCCCAGTGGCAAT	TTGTGTGAAGAAGCCTCGTTG	88
VIM	NM_173969.3	CTCCTACCGCAGGATGTTCG	TGGATGTGGTCACGTAGCTC	140
PECAM1	NM_174571.3	AATCAGAGCGTGGGCTCAAA	ATCCACTGGGGCTATCACCT	147
BMP4	NM_001045877.1	AGCTTCCACCACGAAGAACAT	CACCTCGTTCTCTGGGATGC	102
AFP	NM_001034262.2	CGGACCTTCCGAGCCATAAC	CTCTTTCCCCATCCTGCAGAC	154
FOXA2	XM_025001047.1	CGAGCCCGAGGGCTACTC	GTACGTGTTCATGCCGTTCA	92
mOSKM		ACGAGCCACAAGCTCACCTCT	GGCATTAAAGCAGCGTATCC	221

**Table 2:** Efficiency of reprogramming at D14 (number of colonies observed in relation to number of cells plated) derived in 5% and 20% oxygen tension and cultured with bFGF or bFL2i supplementation.

	5% O2	20% O2	Total
bFGF	0.02%	0.005%	0.0125%
bFL2i	0.075%	0.07%	0.0725%
Total	0.0476%	0.0375%	

## Data Availability

The authors confirm that all data and materials support the published claims and comply with field standards.

## Supplementary Materials

The following are available online at https://www.mdpi.com/article/10.3390/cells10061531/s1, Figure S1: Immunostaining of OCT4 (red) and SOX2 (green) in biPSCs derived in different oxygen tensions (low × high O_2_) and pluripotent supplements (bFGF × bFL2i). The nuclei were stained with Hoechst 33342 (blue). Magnification 10×, scale bars: 200 μm.
