# Supplementary figures and images for: Cattle In Vitro Induced Pluripotent Stem Cells Generated and Maintained in 5 or 20% Oxygen and Different Supplementation
Source: Cells. 2021 Jun 17;10(6):1531. doi: 10.3390/cells10061531 (PMC8234940; doi:10.3390/cells10061531)

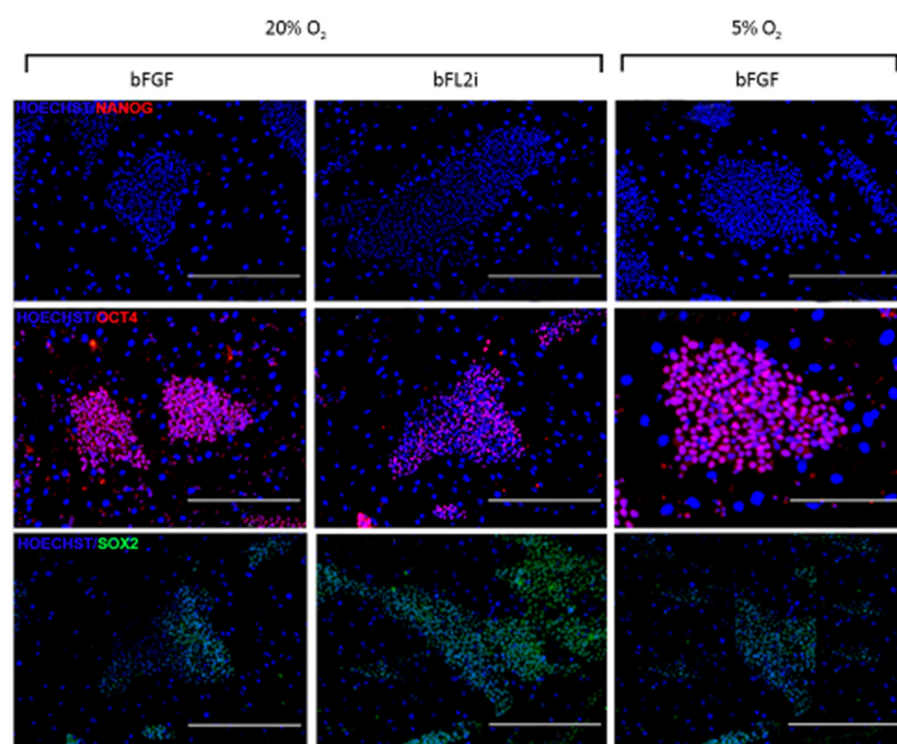

Supplement: Supplementary file 1 [file cells-10-01531-s001.zip › cells-1224961-supplementary.pdf]
